# Redefining mild hypoxic‐ischaemic encephalopathy: A fundamental step to therapeutic progress

**DOI:** 10.1111/dmcn.70225

**Published:** 2026-02-21

**Authors:** Jeanie L. Y. Cheong

**Affiliations:** ^1^ Clinical Sciences, Murdoch Children's Research Institute Parkville Victoria Australia

## Abstract

**This commentary is on the original article by Romeo et al. on pages** 1261–1268 **of this issue.**

Despite the decline in the global incidence of neonatal encephalopathy since 1990, 1.0 million infants are still affected every year.^1^ Therapeutic hypothermia is effective in reducing death or moderate–severe disability in infants with moderate to severe hypoxic‐ischaemic encephalopathy (HIE), but questions remain about its efficacy and safety in infants with mild HIE. Mild HIE is not ‘benign’, with neurodevelopmental disabilities reported in 16% to 25% of survivors.^2^ However, trials focused on mild HIE have yet to identify an effective therapy.

A key problem underpinning trial design for infants with mild HIE is its definition – many rely on a combination of biochemical (evidence of birth acidosis), obstetric sentinel events, ongoing receipt of neonatal resuscitation, Apgar scores, and mild HIE on clinical Sarnat staging. The latter is crude, subjective, and does not account for the neurological evolution of HIE within the critical 6‐hour time window before therapeutic intervention. Thus, neurodevelopmental outcomes for mild HIE are heterogenous. There is a need for better precision to identify infants at highest risk of later neurodevelopmental problems as candidates for neuroprotection trials.

There have been recent attempts to improve early risk stratification within mild HIE. Using data from the PRIME study, Chalak et al.^3^ developed a scoring system using a total numerical Sarnat score from each of the six categories (range 0–18). The total Sarnat score of ≥5 predicted disability at 18 to 22 months with good accuracy by area under the curve (AUC) 0.83, high sensitivity (100%), and fair specificity (67%). Limitations of this study included a small sample size (*n* = 43), and no comparison made with the widely used 3‐stage modified Sarnat score. Interestingly, when applied retrospectively to hypothermia trial participants with moderate to severe HIE, there was little difference in the AUC estimates, sensitivity, and specificity for death or disability between the total numerical Sarnat score and the modified Sarnat staging.^4^ An important point of difference between the studies was that the numerical Sarnat in the latter study did not distinguish between normal (score 0) and mild (score 1). The utility of total Sarnat scores in risk stratification of mild HIE warrants further examination.

Romeo et al.^5^ sought to tighten the definition of mild HIE by including a normal amplitude‐integrated encephalogram (aEEG) to the biochemical and modified Sarnat criteria. All 40 infants had typical neuromotor development and developmental quotients at 12 months, notwithstanding subtle neurological abnormalities on brain magnetic resonance imaging or a standardized neonatal neurological examination noted in 25% of infants. Inclusion of aEEG may help distinguish severity within mild HIE, and aid in selection of a more severe group for neuroprotective trials.

An additional insight gained from Romeo et al.'s study was the natural history of hypotonia, which was noted in 66% of their cohort on initial assessment.^5^ Whilst other studies had identified tone abnormalities within the first 6 hours as an important predictor of later neurodevelopment,^3^ all infants in Romeo et al.'s study had normal standardized infant neurological assessments by 6 months, and normal developmental quotients by 12 months. Hypotonia in the first 6 hours as a discriminating sign for later neurodevelopment needs further exploration.

So, where does this leave us? The current definition of mild HIE is inadequate and may misclassify risk. New scoring systems for encephalopathy severity, and/or addition of early aEEG, show promise. The utility of new definitions for mild HIE need replication in larger cohorts, ideally pooling data from different geographic regions. Only then can we accelerate efforts to seek better neuroprotection therapies for this group of infants.

## Data Availability

Not required.
